# Values of Apparent Diffusion Coefficient and Lesion-to-Spinal Cord Signal Intensity in Diagnosing Solitary Pulmonary Lesions: Turbo Spin-Echo versus Echo-Planar Imaging Diffusion-Weighted Imaging

**DOI:** 10.1155/2021/3345953

**Published:** 2021-08-10

**Authors:** Qiang Lei, Qi Wan, Lishan Liu, Jianfeng Hu, Wei Zuo, Jianneng Li, Guihua Jiang, Xinchun Li

**Affiliations:** ^1^Department of Medical Imaging, Guangdong Second Provincial General Hospital, Shiliugang Rd, Haizhu District Guangzhou, China 510317; ^2^Department of Radiology, The First Affiliated Hospital of Guangzhou Medical University, 151 Yanjiangxi Road, Guangzhou, China 510120; ^3^Department of Radiology, The Fifth Affiliated Hospital of Guangzhou Medical University, 621 Gangwan Road, Guangzhou, China 510799; ^4^Department of Medical Imaging, Guangdong Second Provincial General Hospital, Shiliugang Rd, Haizhu District Guangzhou, China 510317

## Abstract

**Objective:**

This study is aimed at comparing the image quality and diagnostic performance of mean apparent diffusion coefficient (ADC) and lesion-to-spinal cord signal intensity ratio (LSR) derived from turbo spin-echo diffusion-weighted imaging (TSE-DWI) and echo-planar imaging- (EPI-) DWI in patients with a solitary pulmonary lesion (SPL).

**Methods:**

33 patients with SPL underwent chest imaging using EPI-DWI and TSE-DWI with *b* = 600 s/mm^2^ in free breathing. A comparison of the distortion ratio (DR), signal-to-noise ratio (SNR), and contrast-to-noise ratio (CNR) was drawn between the two techniques using a Wilcoxon signed-rank test. The interprotocol reproducibility between quantitative parameters of EPI-DWI and TSE-DWI was evaluated using a Bland-Altman plot. ADCs and LSRs derived from EPI-DWI and TSE-DWI were calculated and compared between malignant and benign groups using the Mann–Whitney test.

**Results:**

TSE-DWI had similar SNR and CNR compared with EPI-DWI. DR was significantly lower on TSE-DWI than EPI-DWI. ADC and LSR showed slightly higher values with TSE-DWI, while the Bland-Altman analysis showed unacceptable limits of agreement between the two sequences. ADC and LSR of both DWI techniques differed significantly between lung cancer and benign lesions (*P* < 0.05). The LSR_(EPI-DWI)_ showed the highest area under the curve (AUC = 0.818), followed by ADC_(EPI-DWI)_ (AUC = 0.789), ADC_(TSE-DWI)_ (AUC = 0.781), and LSR_(TSE-DWI)_ (AUC = 0.748), respectively. Among these parameters, the difference in diagnostic accuracy was not statistically significant.

**Conclusions:**

TSE-DWI provides significantly improved image quality in patients with SPL as compared with EPI-DWI. However, there was no difference in diagnostic efficacy between these two techniques, according to ADC and LSR.

## 1. Introduction

Diffusion-weighted imaging (DWI) is an essential magnetic resonance imaging (MRI) technique to assess water molecule diffusivity in living tissues. Single-shot echo-planar imaging (SS-EPI) is the most used DWI technique because of its short acquisition time [1]. However, the image quality of the standard SS-EPI-DWI is frequently deteriorated by magnetic inhomogeneity, as EPI acquisition is prone to phase error accumulation [2].

In the lung, magnetic inhomogeneity exists at the air-lung interface, leading to signal loss or image distortion in EPI-DWI, which might hamper accurate lesion measurements of the derived parameters [3]. Turbo spin-echo (TSE-) DWI might be an excellent alternative to EPI-DWI for patients sensitive to image distortion [4–6]. In recent years, studies [7–9] compared the image quality and the reproducibility of apparent diffusion coefficient (ADC) between EPI-DWI and TSE-DWI in areas such as oral, head, and neck. A recent study has documented the ability of TSE-DWI to improve the image quality and test-retest robustness of ADC and intravoxel incoherent motion (IVIM) parameters in patients with lung cancer [10]. However, it is unclear whether there is any diagnostic difference between these two sequences in assessing solitary pulmonary lesions.

Previous studies suggested that ADC could be a useful parameter in distinguishing malignant from benign pulmonary lesions [11]. On the other hand, some studies demonstrated that the lesion-to-spinal cord signal intensity ratio (LSR), as a semiquantitative method, has better diagnostic efficacy than ADC [12]. However, other studies reported inconsistent findings [13–15].

Therefore, the purpose of this study was to compare the image quality as well as the diagnostic performance of ADC and LSR derived from EPI-DWI and TSE-DWI in patients with solitary pulmonary lesions (SPLs).

## 2. Materials and Methods

### 2.1. Patient Population

This study was approved by the institutional review board, and written informed consent was obtained from each patient with a solitary pulmonary nodule or masses confirmed by computed tomography (CT) findings. The inclusion criteria were as follows: (a) lesions were measurable on CT images; (b) there were no contraindication for MRI examinations; and (c) patients did not undergo any therapies. A total of 33 patients (21 men and 12 women; age range, 33–77 years; mean age, 57 years) underwent a DWI scan of the lung. The pathological assessment revealed 22 malignant tumors (15 invasive adenocarcinomas, one squamous carcinoma, three small-cell carcinomas, one lymphoid epithelioid carcinoma, and two mucoepidermoid carcinomas) and 11 benign lesions (one hamartoma, two sclerosing alveolar cell tumors, and eight granulomas).

### 2.2. Image Acquisition

All patients were examined on a 3.0 T MRI scanner equipped with a 16-channel body phase array coil (Achieva, Philips Healthcare, Best, the Netherlands). Axial TSE T2-weighted imaging (T2WI) was performed with the following parameters: repetition time (TR), 973 ms; echo time (TE), 80 ms; field of view (FOV), 430 × 349 mm^2^; matrix size, 360 × 247; and slice thickness, 7 mm, with a 0.7 mm intersection gap. Both SS-TSE sequence and SS-EPI sequences with *b*_0_ and *b* value 600 s/mm^2^ were used to acquire DWI images in free breathing. The EPI-DWI's sequence parameters were as follows: FOV, 260 × 423 mm^2^; TR/TE, 1238 ms/51 ms; slice thickness, 5 mm; number of signal averages (NSA), 3; and acquisition time, 36 s. The TSE-DWI's sequence parameters were as follows: FOV, 260 × 423 mm^2^; TR/TE, 5965 ms/56 ms; slice thickness, 5 mm; NSA, 3; and acquisition time, 2 min 47 s (Table 1).

### 2.3. Image Quality

The image quality of 33 patients was quantitatively assessed by a radiologist under an experienced radiologist (five years and ten years of magnetic resonance diagnostic practice, respectively). The image distortion along a phase-encoding direction, signal-to-noise ratio (SNR), and contrast-to-noise ratio (CNR) from EPI-DWI and TSE-DWI (with *b* = 600 s/mm^2^) were recorded. Regions of interest (ROIs) were manually drawn on the solid part of the lesion at the level of maximum transverse diameter avoiding necrosis and hemorrhage, spinal cord (12–16 mm^2^), thoracic muscle (40–50 mm^2^), and background (40–50 mm^2^) for mean signal intensity (SI) and standard deviation (SD) measurements.

In the RadiAnt DICOM Viewer 4.6.5.18450 (Medixant, Poznan, Poland), the fusion images were generated by superimposing DWI with T2WI in each sequence, and image distortions of the solitary pulmonary nodules or mass were evaluated using the distortion ratio (DR):

(1) $$\begin{array}{c} \text{DR}={\frac{a}{b}}^{10}, \end{array}$$

where *a* is the maximum displacement along the phase-encoding path of the lesion between T2WI and the corresponding DWI and *b* is the diameter along the phase-encoding path of the lesion on T2WI.

SNR, CNR, and LSR were calculated according to the following equations:

(2) $$\begin{array}{c} \text{SNR}={\frac{{\text{SI}}_{\text{lesion}}}{{\text{SI}}_{\text{background}}}}^{16},\\ \text{CNR}={\frac{{\text{SI}}_{\text{lesion}}-{\text{SI}}_{\text{muscle}}}{{\text{SD}}_{\text{background}}}}^{17},\\ \text{LSR}={\frac{{\text{SI}}_{\text{lesion}}}{{\text{SI}}_{\text{spinal cord}}}}^{12}. \end{array}$$

### 2.4. Image Postprocessing

MRI images were postprocessed using PRIDE software (Philips Healthcare). ADC was generated with *b* values of 0 and 600 s/mm^2^. ROIs were manually drawn in the solid lesion on the slice with a maximum transverse diameter under the guidance of an experienced radiologist, avoiding necrosis and hemorrhage.

The ADC value was calculated using a monoexponential fit of signal intensity according to the following equation:

(3) $$\begin{array}{c} \frac{S\left(b\right)}{S_{0}}=\exp\left(-b\text{ADC}\right), \end{array}$$

where *S*(*b*) and *S*_0_ are the diffusion-weighted signal intensity at a given *b* value and *b* = 0 s/mm^2^, respectively. The least-squares method was used for linear fitting of the monoexponential model.

### 2.5. Statistical Analysis

Normally distributed and nonnormally distributed data were presented as the mean (SD) and median (IQR), respectively. DR, SNR, and CNR were compared between EPI-DWI and TSE-DWI using the Wilcoxon signed-rank test. The interprotocol reproducibility of ADC and LSR between the two sequences was assessed by calculating the 95% Bland-Altman limits of agreement (LOAs). The ADC and LSR were evaluated using the Mann–Whitney test and receiver operating characteristic (ROC) analysis. Statistical analyses were carried out using SPSS (version 22.0, IBM Corporation, USA) and MedCalc software (version 18.2.1, Mariakerke, Belgium). A *P* value < 0.05 was considered statistically significant.

## 3. Results

### 3.1. Image Quality

**Figure 1:**
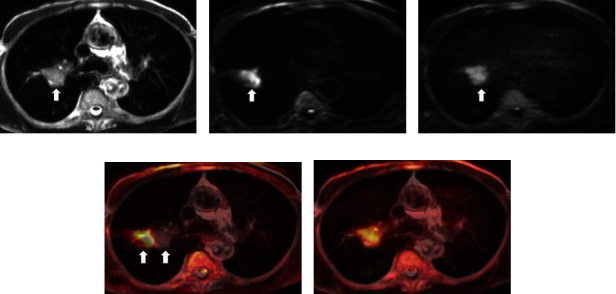
A nodular lesion (arrow) in the right upper lobe: (a) TSE-T2WI; (b) EPI-DWI (*b* = 600 s/mm^2^); (c) TSE-DWI (*b* = 600 s/mm2); (d) T2WI fused with EPI-DWI; (e) T2WI fused with TSE-DWI. The distortion and displacement of the lesion (double fine arrow) are illustrated on EPI-DWI. Fused images demonstrated that TSE-DWI is perfectly matched with T2WI.

**Figure 2:**
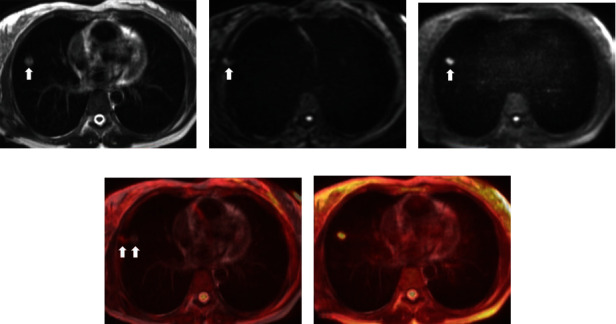
A nodular lesion (arrow) in the right middle lobe: (a) TSE-T2WI; (b) EPI-DWI (*b* = 600 s/mm^2^); (c) TSE-DWI (*b* = 600 s/mm^2^); (d) T2WI fused with EPI-DWI; (e) T2WI fused with TSE-DWI. The distortion of the lesion (double fine arrow) is illustrated on EPI-DWI, while the lesion on TSE-DWI demonstrates node formation or displacement compared to T2WI.

**Figure 3:**
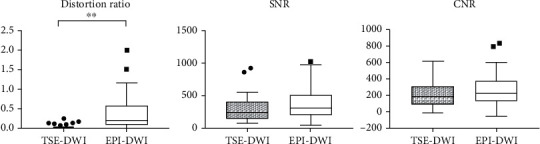
Comparison of distortion ratio (DR), signal-to-noise ratio (SNR), and contrast-to-noise ratio (CNR) between SS-TSE-DWI and SS-EPI-DWI. Significant differences in DR were evaluated with a Wilcoxon signed-rank test. ∗∗ denotes *P* < 0.001.

Two representative fusion images, consisting of TSE-DWI on T2WI and EPI-DWI on T2WI, are shown in Figures 1 and 2, respectively.  Figure 3 shows a contrast of DR, SNR, and CNR between EPI-DWI and TSE-DWI. Image distortion of DWI was significantly reduced in TSE-DWI as contrasted to EPI-DWI. The DR for SPLs in TSE-DWI and EPI-DWI was 0.000 (0.029) and 0.224 (0.483), respectively (*P* < 0.001). The mean SNR and CNR were slightly lower in TSE-DWI than those in EPI-DWI; however, no significant difference was detected between the two sequences.

### 3.2. Comparison of ADC and LSR between TSE-DWI and EPI-DWI

**Figure 4:**
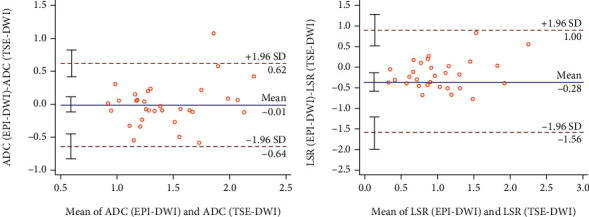
Bland-Altman plots for LSR and ADC of EPI-DWI and TSE-DWI. Continuous blue lines demonstrate mean differences, and dotted red lines show 95% limits of agreement (LOA).

Figure 4 shows the Bland-Altman plots of ADC and LSR between EPI-DWI and TSE-DWI. The ADC and LSR of TSE-DWI exhibited slightly greater values than those of EPI-DWI in solitary pulmonary lesions (SPLs). The Bland-Altman analysis illustrated broad LOA between EPI-DWI and TSE-DWI. The 95% LOAs obtained were from -64.00% to 62.00% for ADC and from -156.00% to 100.00% for LSR.

### 3.3. Diagnostic Performance of Multiple Parameters

**Figure 5:**
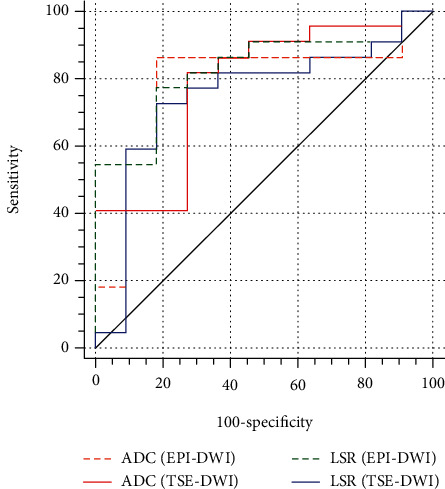
Results of receiver operating characteristic analysis for LSR and ADC derived from TSE- and EPI-DWI.

The ADC and LSR derived from EPI-DWI and TSE-DWI of malignant and benign SPLs are shown in Table 2, and the corresponding ROC curves for predicting malignant SPLs are plotted in Figure 5. The area under the curve (AUC) of ADC derived from TSE-DWI and EPI-DWI was 0.781 and 0.789, respectively, and the corresponding cutoff value, sensitivity, and specificity were 1.450, 81.82%, and 72.73% and 1.380, 86.36%, and 81.82%, respectively. Furthermore, the AUC of LSR derived from TSE-DWI and EPI-DWI were 0.748 and 0.818, respectively, and the corresponding cutoff value, sensitivity, and specificity were 1.060, 72.73%, and 81.82% and 0.878, 77.27%, and 81.82%, respectively (Table 3). However, no significant differences in diagnostic efficacy were observed among ADC_(TSE-DWI)_, ADC_(EPI-DWI)_, LSR_(TSE-DWI)_, and LSR_(EPI-DWI)_.

## 4. Discussion

In the present study, we found that TSE-DWI provides distortion-free images with similar SNR and CNR compared with EPI-DWI. The diagnostic performance of ADC and LSR derived from TSE-DWI was as good as those from EPI-DWI. Additionally, LSR showed similar diagnostic performance compared to ADC.

The results of this study show that TSE-DWI may be an excellent substitute for EPI-DWI in patients undergoing lung examinations. As demonstrated by the DR comparison along the phase-encoding direction, free-breathing SS-TSE-DWI was superior to free-breathing EPI in minimizing geometric distortions. In TSE-DWI, the geometric precision was compared with that of a standard anatomic T2WI. Minimum distortion allowed easy registration and transfer of contours between T2WI and TSE-DWI. In EPI-DWI, a shift in the lesion position and distortion was often present at the lesion-air interface due to susceptibility artifacts.

The image noise lies in the voxel size, receiver bandwidth, and the total number of averages during image acquisition [16]. In this study, the two DWI sequences had identical voxel sizes and number of averages. Although the larger inherent bandwidth of the TSE sequence could reduce SNR [3, 8], the SNR and CNR of the two sequences were identical; this finding is similar to a previous research [10].

The Bland-Altman analysis demonstrated broad LOAs for ADC (64%) between EPI-DWI and TSE-DWI in our study, similar to previous studies [10, 17]. Moreover, we found that the LoA for LSR (up to 156.00%) between the two sequences was even broader than that for ADC. This further confirms that the parameters of these two sequences cannot be directly substituted for each other. Consequently, the use of a specific quality assurance protocol as that proposed in the recent paper [18] is deeply recommended, in order to guarantee the clinical applications of the parameters of these two sequences.

In our study, we found that both EPI-DWI and TSE-DWI can distinguish malignant from benign SPLs according to ADC and LSR. The ADC in the malignant was lower than that in benign lesions, and LSR showed the opposite result. This is because malignant lesions tend to have a higher cell density and narrower extracellular space, which hinders the diffusion movement of water molecules [19]. Besides, we compared LSR and ADC from both sequences in the differentiation of SPLs and found no significant difference between them. Our results are consistent with some studies [13, 20], but not the same as others [12, 21]. These inconsistent results are likely to be attributed to the bias of the included cases, lesion size, variance in imaging quality, and the reduced sample size.

Unexpectedly, even with less image distortion, the quantitative parameters of ADC and LSR derived from TSE-DWI are not better than (or even slightly second to) EPI-DWI in terms of diagnostic accuracy. LSR_(TSE-DWI)_ showed the lowest diagnostic efficiency, although the difference among these parameters was not significant. One possible explanation is that ROI was typically placed at the location with the highest signal intensity during assessing LSR. The acquisition time of TSE-DWI is longer, and its blurring effect may affect the ROI delineation and interpretation of lesions' signal intensity. This should be confirmed in future studies.

This study had some limitations. First, the image acquisition times of EPI-DWI and TSE-DWI were not the same. Therefore, the SNR per unit time was not the same. We did not increase the acquisition time of EPI to the same as that of TSE, which is what we are going to do in our following studies; this could lead to a significantly higher SNR and CNR for EPI compared to TSE sequences. Second, the population of this study was relatively small. Some other benign (e.g., round atelectasis) and malignant (e.g., carcinoid and single metastasis) lesions were not included in this study. Furthermore, only nodules larger than 8 mm in diameter and pathologically confirmed were included; the inclusion of all nodules encountered in clinical practice might yield different results.

In conclusion, TSE-DWI can be an excellent alternative to EPI-DWI for imaging pulmonary lesions sensitive to distortion. Despite less image distortion, the quantitative parameters of ADC and LSR from TSE-DWI demonstrate similar diagnostic accuracy for the differentiation of SPLs as compared with EPI-DWI.

## Figures and Tables

**Table 1:** Scanning parameters of magnetic resonance imaging.

	TSE-T2WI	EPI-DWI	TSE-DWI
TR (ms)	973	1238	5965
TE (ms)	80	51	56
NSA	1	4	4
FOV (mm)	430 × 349	260 × 423	260 × 423
Slice thickness (mm)	7	5	5
Gap (mm)	0.7	0.5	1
*b* value (s/mm^2^)	—	600	600
Parallel imaging factor	2	3	3
Matrix	360 × 247	88 × 140	80 × 140
BW (Hz/pixel)	565.2	43.5	371.4
Recon voxel size (mm)	0.67	1.1	1.1
Scanning time	23 s	36 s	2 min 47 s

Note: TE: echo time; TR: repetition time; NSA: number of signals acquired; FOV: field of view; m: minutes; s: seconds; BW: bandwidth.

**Table 2:** The mean ADC and LSR of TSE-DWI and EPI-DWI.

Parameters	Benign	Malignant	*Z*	*P*
*TSE*				
ADC (×10^−3^ mm^2^/s)	1.700 (0.725)	1.326 (0.366)	-2.598	0.009
LSR	0.757 (0.354)	1.304 (0.780)	-2.291	0.022
*EPI*				
ADC (×10^−3^ mm^2^/s)	1.600 (0.616)	1.229 (0.327)	-2.673	0.008
LSR	0.603 (0.386)	1.073 (0.471)	-2.941	0.003

Note: data as median (IQR); Mann–Whitney test.

**Table 3:** Sensitivity and specificity of ADC and LSR at optimal cutoff values in differentiating malignant from benign solitary pulmonary lesions.

Parameters	AUC	Cutoff value	Sensitivity (%)	Specificity (%)	+LR (%)	-LR (%)
*TSE*						
ADC (×10^−3^ mm^2^/s)	0.781	1.450	81.82	72.73	3.00	0.25
LSR	0.748	1.060	72.73	81.82	4.00	0.33
*EPI*						
ADC (×10^−3^ mm^2^/s)	0.789	1.380	86.36	81.82	4.75	0.17
LSR	0.818	0.878	77.27	81.82	4.25	0.28

Note: AUC: area under the ROC curve; ADC: apparent diffusion coefficient; LSR: lesion-to-spinal cord signal intensity ratio; +LR: positive likelihood ratio; -LR: negative likelihood ratio.

## Data Availability

All the materials and data in studies are available.
